# A new era for RSV: the end in sight?

**DOI:** 10.1016/j.lana.2025.101364

**Published:** 2026-01-09

**Authors:** The Lancet Regional Health – Americas

Respiratory Syncytial Virus (RSV) is a leading cause of lower respiratory tract infections in infants and young children worldwide, causing approximately 3.6 million hospitalisations and 100,000 deaths annually in children younger than 5 years. This scenario, however, is set to change dramatically in the coming years with maternal vaccination and monoclonal antibodies. Yet, two challenges remain: timing and access.

Maternal RSV immunisation was designed around temperate winter peaks, but seasonality across Central and South America means a fixed vaccination window might miss local surges. For example, RSV activity in Chile clusters in winter but varies year to year, while Brazil's regional differences make a single national schedule inadequate. While the seasonal vaccination schedule works for the USA and Canada, effective maternal immunisation in Central and South America will require granular surveillance data to design the best programmes for each territory. Year-round deployments can be a solution for areas with undefined or variable seasonality, but costs and logistics might be inhibitive. A Brazilian cost-effectiveness study confirmed that the vaccine's impact would only be viable if priced at 30% of the manufacturer's suggestion. Brazil recently secured a technology transfer agreement for the local production of RSV vaccine, which will lower costs and support access through the public immunisation programme for all pregnant people, in place since December, 2025, but the schedule and access for other countries within Latin America and the Caribbean remain undefined.

Current maternal immunisation is restricted to the RSVpreF vaccine (Abrysvo), administered between 32 and 36 weeks of pregnancy. The short safety window limits its use to regions and communities with strong antenatal coverage. This remains a distant reality for many low-income and middle-income countries (LMICs) across Latin America and the Caribbean, and for rural or systematically excluded communities where antenatal care is inadequate. As a silver lining, the time-sensitive nature of this strategy can prompt health systems to revisit and strengthen antenatal care, widening access and improving continuity of care.

For unvaccinated newborns, monoclonal antibodies at birth can provide early protection during the highest-risk period. Nirsevimab, included in WHO recommendations, has shown high effectiveness with a single dose. In Chile, universal at-birth nirsevimab coverage led to major reductions in infant RSV outcomes during the first season. However, the high cost of monoclonal antibodies remains a barrier. While Chile was able to include at-birth access for its entire population, this is less likely to be a reality for LMICs across the region. A promising workflow involves making maternal wards a central point for offering maternal immunisation during antenatal care and embedding birthroom nirsevimab for when immunisation is missed and for high-risk cases such as preterm and chronic conditions, reducing costs. A simulation study in Canada suggests that this combined strategy could reduce infant mortality by 76–85%. Brazil announced the implementation of this strategy with the inclusion of nirsevimab in the public health system starting in February, 2026, restricted to preterm births and newborns with chronic conditions. Meanwhile, the Pan American Health Organization is exploring pooled procurement for price negotiations to reduce costs for nirsevimab, but in the absence of such agreements, access will remain limited in most countries of the region.

Prevention of RSV-related newborn deaths is within reach, but will require national programmes to ensure strong antenatal care that supports effective vaccination programmes and sustained access to vaccines for all pregnant people, and monoclonal antibodies for high-risk and unvaccinated newborns. Internal assessments will be key to understanding capacity and adapting the strategy that can sustainably deliver the greatest benefit. The path is unequal among countries. While the USA, Canada, and more recently Brazil and Chile are quickly advancing in the implementation of these new preventive strategies, most countries in the Americas still do not have a national programme for RSV immunisation or anticipate procurement for monoclonal antibodies. These contrasting realities are a representative image of the most unequal region in the world.

Maternal vaccination and monoclonal antibodies promise to transform RSV prevention and save millions of lives, but how do we ensure equity in access and sustainable production, distribution, and delivery for all? The rapid pace of vaccine innovation has outstripped investment in prenatal and child health infrastructure. Equalising access for sustained availability across the heterogeneous Americas region will define success in preventing RSV. The challenge ahead is not only to deploy these innovations, but also to build a health-care system that can deliver them equitably and sustainably so that every child can benefit from this new era in RSV prevention.

